# Bottlenecks, concerns and needs in malaria operational research: the perspectives of key stakeholders in Nigeria

**DOI:** 10.1186/s13104-018-3379-5

**Published:** 2018-05-04

**Authors:** Pamela Onyiah, Al-Mukhtar Y. Adamu, Rotimi F. Afolabi, Olufemi Ajumobi, Maduka D. Ughasoro, Oluwaseun Odeyinka, Patrick Nguku, IkeOluwapo O. Ajayi

**Affiliations:** 1 Nigeria Field Epidemiology and Laboratory Training Programme, Abuja, Nigeria; 2 Department of Medical Microbiology and Parasitology, Faculty of Clinical Sciences, College of Health Sciences, Bayero University, Kano, Nigeria; 3 Department of Epidemiology and Medical Statistics, Faculty of Public Health, College of Medicine, University of Ibadan, Queen Elizabeth Road, UCH Campus, Ibadan, 23402 Oyo State Nigeria; 4 African Field Epidemiology Network, Abuja, Nigeria; 5 Department of Paediatrics, University of Nigeria Enugu Campus, Enugu, Nigeria

**Keywords:** Malaria, Bottlenecks, Stakeholders, Operational research, Nigeria

## Abstract

**Objective:**

We conducted a study to determine stakeholders’ perspective of the bottlenecks, concerns and needs to malaria operational research (MOR) agenda setting in Nigeria.

**Results:**

Eighty-five (37.9%) stakeholders identified lack of positive behavioural change as the major bottleneck to MOR across the malaria thematic areas comprising of malaria prevention 58.8% (50), case management 34.8% (39), advocacy communication and social mobilisation 4.7% (4) while procurement and supply chain management (PSM) and programme management experts had the least response of 1.2% (1) each. Other bottlenecks were inadequate capacity to implement (13.8%, n = 31), inadequate funds (11.6%, n = 26), poor supply management (9.4%, n = 21), administrative bureaucracy (5.8%, n = 13), inadequacy of experts (1.3%, n = 3) and poor policy implementation (4.9%, n = 11). Of the 31 stakeholders who opined lack of capacity to execute malaria operational research; 17 (54.8%), 10 (32.3%), 3 (9.7%) and 1 (3.2%) were experts in case management, malaria prevention, surveillance, monitoring and evaluation and PSM respectively. Improvement in community enlightenment and awareness strategies; and active involvement of health care workers public and private sectors were identified solutions to lack of positive behavioural change.

**Electronic supplementary material:**

The online version of this article (10.1186/s13104-018-3379-5) contains supplementary material, which is available to authorized users.

## Introduction

Globally, malaria has been reported as the most deadly and life-threatening parasitic disease [1]. In 2015, an estimated 212 million new cases of malaria and 429,000 deaths were recorded globally; African region accounted for about 90 and 92% of these respectively [2]. Nigeria accounted for 29% of malaria global burden and 26% of its global deaths [2].

Though the incidence rate of malaria has decreased by 41% globally between 2000 and 2015, malaria continues to have a devastating impact and efforts must be accelerated for reduction of the incidence rate especially in African region [2]. Thus, World Health Organisation (WHO) has emphasised the importance of operational research (OR) and setting research priorities that can help direct research towards malaria elimination specific needs [3, 4]. It is expected that OR significantly contributes to influencing policy change or improving performance at all levels [5]. Operational research, an integral part of disease control programme activities is important in providing an evidence-base for context-specific implementation of global best-practice interventions to maximise their outcome and impact, and identifying hindrances to programme performance [6, 7]. Locally, OR aims to harness the relevant answers and solutions that can be used by a specific programme based on the setting’s peculiarities [6, 7].

In sub-Saharan Africa, access to prevention and case management (CM) intervention is sub-optimal [2]. Operational research studies are meant to assess the feasibility of new interventions in country specific settings, identify bottlenecks in malaria control [8, 9] and are essential for evaluation of integration of new methods into routine health systems to improve malaria diagnosis and outcomes [3]. A huge bottleneck exists which varies by region despite significant improvements in access to core malaria control interventions [2].

In the last decade, majority (77%) of malaria OR (MOR) projects implemented globally were from WHO Africa region countries—Nigeria ranked 3rd (Tanzania 1st and Kenya 2nd) [9]. Meanwhile, prioritisation of MOR for effective malaria control and eventual elimination is a clearly outlined strategy in the National Malaria Strategic Plan (NMSP) [10, 11]. The Nigeria National Malaria Elimination Programme (NMEP) held MOR stakeholder workshops in 2010, 2012, 2013, 2014 and produced a list of harmonised MOR questions prioritised by relevance and thematic areas. Only three (9%) of 33 prioritised questions were answered between 2014 and 2016. This very low uptake of MOR questions is a cause for concern and recognizes the need to unravel probable bottlenecks militating against answering these questions [12]. Therefore, we elicited stakeholders’ perspectives on bottlenecks, concerns and needs of MOR agenda setting in Nigeria as part of preliminary study towards setting a National MOR agenda.

## Main text

### Methods

#### Study design and population

The NMEP formed and led a planning committee for country MOR dialogue comprising thematic program officers and other malaria control partners including Nigeria Field Epidemiology and Laboratory Training Programme (NFELTP) [11]. The NMEP initiated the research. The NFELTP was assigned the responsibility of setting up and leading a MOR Agenda—Setting Task Team which conducted a preliminary study towards identification of OR gaps, bottlenecks and needs.

A cross-sectional study was conducted in Nigeria from October to November 2016. The study population was malaria researchers in and outside Nigeria who must have conducted or involved in malaria research in Nigeria within the last decade. They were identified from publications and snowballing. The search was conducted with keywords’ combination of ‘malaria, agenda setting, operation research and priorities’ using Pubmed, Web of Science, Google Scholar and Medline. Recent publications from 2010 to 2016 were included. The participants comprised of development partners, non-governmental organisation stakeholders, malaria policy makers and implementers at all tiers of government, healthcare workers and malaria research experts in the academia.

#### Data collection and analysis

A semi-structured questionnaire was adapted from a previous study [13] and pretested among nine malaria stakeholders in Abuja-Nigeria prior to the survey. The questionnaire was revised by the 12-member MOR agenda setting task-team [11, 14] comprising NFELTP fellows, NMEP officers and university researchers. The same earlier validated questionnaire was self-administered using both paper-based and online survey platform. The online survey was administered using Survey Monkey^®^ to ensure participation of malaria researchers who were unavailable in Nigeria or were residents elsewhere globally. The questionnaire sought information on personal biodata, area of expertise, experience in MOR in Nigeria, challenges, bottlenecks in MOR and needs to address the gaps identified from the participants (Additional file 1). The responses to bottlenecks and needs were grouped into themes and rated using a score of 1–5 based on priority in MOR with 1 being most priority and 5 least in priority. Data were entered and cleaned using Microsoft excel version 2007. IBM SPSS version 20 was used for data analysis. Results based on themes in malaria control were presented in frequencies and proportions.

### Results

#### Socio-demographic characteristics of the study participants

Of 224 identified researchers, 185 (82.6%) participated in the study out of which 85 (45.9%) participated in the face-to-face survey and 100 (54.1%) of the participants did the survey “Online”. The mean age of the participants was 45.0 years (standard deviation: 9.1), and 125 (70.6%) were males. Highest proportion of the participants were malaria policy-makers and programme managers (21.1%); parasitologist (16.7%) and clinicians (15.6%) (Table 1).

**Table 1:** Socio-demographic characteristics of the study participants

Characteristics	n	%
Mode of administration of questionnaire (*N *= 185)		
Online	100	54.1
Face to face	85	45.9
Sex (*N *=177)		
Male	125	70.6
Female	52	29.4
Area of expertise (*N *=180)		
Policy and programme management	38	21.1
Parasitology	30	16.7
Clinical management	28	15.6
Data management	19	10.6
Therapeutics	13	7.2
Entomology	12	6.7
Public Health	11	6.1
Medical laboratory	10	5.6
Administrator	6	3.3
Health education and communication	3	1.7
Medical sociology	3	1.7
Others (researcher)	7	3.9

#### Working experience of the study participants on MOR and its associated challenges

Majority (94.9%) of the participants’ work experience were currently Nigeria-based; while only 19.8% of them had malaria work experience outside Nigeria. About half of the respondents (50.8%) opined that Nigeria’s MOR agenda should be different from that of other settings. Almost half (48.6%) of the participants had been involved in MOR in Nigeria, of which 43.7% had expertise in CM, 40.8% in malaria prevention (MiP) and 1.4% in Advocacy, Communication and Social Mobilisation (ACSM). Most (72.2%) participants had challenges while conducting MOR: frequent among them were “poor community participation/attitude” (35.5%), “poor commodity supply” (32.3%) and “poor funding” (32.3%) (Additional file 2).

#### Malaria operational research concerns and needs as presented by the stakeholders

Stakeholders from all expertise believed bottlenecks were hindering MOR in Nigeria. Lack of positive behavioural change was observed as a bottleneck by the stakeholders in all the malaria thematic areas comprising MiP, CM, procurement and supply chain management (PSM), programme management (PM) and ACSM. Of 85 responses got from stakeholders who identified lack of positive behavioural change as a bottleneck, 50 (58.8%) were experts in MiP, while 29 (34.1%) and 4 (4.7%) were experts in CM and ACSM respectively. Only experts in MiP, CM and programme management view inadequate funding as a bottleneck. Lack of competent capacity to execute malaria interventions was also identified as a bottleneck in 4 thematic areas comprising CM (54.8%), MiP (32.3%), Surveillance, Monitoring & Evaluation (9.7%) and PSM (3.2%), having the least response. Poor supply management systems was identified by stakeholders in MiP, CM and PSM thematic groups as a bottleneck impeding MOR execution and implementation. Other bottlenecks identified include administrative bureaucracy, poor implementation of policy and unharmonised coordination and implementation of multiple interventions (Table 2).

**Table 2:** Distribution of MOR bottlenecks identified by respondents in malaria thematic areas (N = 224)

Identified bottlenecks^a^	Number of experts in malaria thematic areas^#^						
	MiP	CM	SME	ACSM	PSM	PM	Total (%)
Lack of positive behavioural change	50	29		4	1	1	85 (37.9)
Inadequate funding	18	2				6	26 (11.6)
Poor supply management	3	15			3		21 (9.4)
Inadequate number of experts	3						3 (1.3)
Poor implementation of policy	6					5	11 (4.9)
Manpower gaps/lack of capacity	10	17	3		1		31 (13.8)
Administrative bureaucracy	7			1		5	13 (5.8)
Unharmonised coordination and implementation of multiple interventions	1					1	2 (0.9)

^a^Multiple responses

^#^Key: *MiP* malaria prevention, *CM* case management, *SME* surveillance, monitoring & evaluation, *ACSM* advocacy, communication & social mobilisation, *PSM* procurement & supply chain management, *PM* programme management

The stakeholders also presented concerns on the effects of the identified bottlenecks in MOR in Nigeria. In most cases the concerns raised were multi-dimensional due to the peculiarities of the thematic areas. Some solutions as to what need to be done were also provided by the experts which were linked to the identified bottlenecks (Table 3). The concerns raised by the stakeholders in respect to behavioural change was that people at the community, government and private levels were resistant to positive changes especially for new programmes. Improvement in community enlightenment and awareness strategies is important for the government to curtail this impediment. Public and private health care workers also need to be actively involved in new programmes and interventions. The major concerns presented for inadequate funding of MOR include lack of budgetary allocation and inadequate leveraging of the limited funds. The stakeholders agreed that identifying domestic fund sources and prioritising areas to disburse the funds would effectively reduce this challenge at national, state and local government area levels (Table 3).

**Table 3:** Concerns and needs linked to the identified bottlenecks in MOR agenda as presented by stakeholders

Bottlenecks	Concerns	Needs
Lack positive behavioural change to malaria interventions	People are resistant to change	Improved community enlightenment and awareness to national programmes
Inadequate funding	Lack of adequate budgetary provision Some sub-themes get more funds than others Unbudgeted miscellaneous	Identification of domestic funding that will enhance national priorities Clarification of all funding sources and amount of funds needed for thematic areas Budgeted research funding at national, state and LGA levels Adequate fund distribution
Poor supply management system	Deterioration and wastage of supplies Delay in supplies Inaccessibility of malaria commodities	Effective logistic supply and management system on malaria commodities. Alternative models for commodity distribution Timely microplanning
Inadequate number of experts	Poor inter-sectorial collaboration for research Lack of experts in the field	Implementation of policy on research interest Technical support
Poor implementation of policy	Inconsistency in following up policy implementation	Increased participation for all stakeholder Enactment of policies turn into law
Manpower gaps/lack of capacity	Health providers attitude	Training on cost-effectiveness Capacity development
Administrative bureaucracy	Conflict of interest Depleted finance Political will	Technical assistance Sector-wide approach with involvement of other line ministries and agencies such as Ministry of environment, regional and urban Planning Dedicated partnership with assigned roles
Unharmonised coordination and implementation of multiple interventions	Interventions not holistic	Multi and trans-disciplinary collaboration Enactment of policies to harmonise interventions

Concerns observed for poor supply management system include wastages or delay in malaria commodities supplies and unnecessary inaccessibility of the commodities. Timely microplanning and effective logistic supply management using alternative models were presented as requirements to curtail this bottleneck. Experts in MiP and PM raised concerns regarding administrative bureaucracy to include conflict of interests and lack of political will to implement MOR. Dedication in partnership and involvement of relevant government sectors was seen as a need to tackle and reduce administration bureaucracy (Table 3).

### Discussion

This study revealed that malaria stakeholders encounter bottlenecks while conducting MOR across thematic areas of malaria control. These had been previously observed in Tanzania during a research priority writing workshop for experts [15].

Lack of positive behavioural change was identified by majority of the stakeholders as a bottleneck; this is very vital as both the community (towards whom the malaria interventions are targeted) and the healthcare workers (who directly implement the intervention) must show commitment and determination in executing such interventions. They must also collaborate with policy-makers in allowing ways to improve the intervention through the appropriate research [16]. Misconceptions due to certain cultural norms or unsubstantiated knowledge to certain interventions such as use of long lasting insecticidal net, artemisinin-based combination therapy or insecticides have been found to hinder the implementation and thereby pose a bottleneck to conduct of research which investigate the interventions’ efficacy let alone allow for their improvement [17, 18].

The poor community participation in MOR may be attributed to lack of awareness and wrong perception of malaria interventions in the community. For malaria intervention programmes to succeed there must be full community involvement as an integral component of the malaria elimination [19].

Inadequate funding is an impediment to the conduct of MOR. The limited fund offered mainly by external donors is not enough to investigate the myriad of problems encountered while executing various malaria interventions. Often research studies are abandoned or inconclusive due to insufficient funds as most of the OR carried out in Nigeria are donor fund-driven [20]. Taking heed of the dwindling international funds especially from the year 2011 [21] which has grossly affected interventions and OR progress in low-income countries, other sources of funds to conduct MOR are required. Some stakeholders opined that domestic sources of fund need to be identified so that focus will be on national priorities for MOR. Likewise, judicious use of the limited fund by leveraging to cover all thematic areas based on the country specific research needs is important to avoid disproportionate allocation of funds to few NMEP thematic areas such as MiP; CM and the neglect of other thematic areas such as OR and PSM [9].

A comprehensive capacity development strategy is required to bridge the manpower and expertise gaps in conducting MOR. Researchers and experts in-country could develop a cost-effective training programme for young researchers which can be anchored by NMEP, NFELTP or similar malaria oriented bodies [16].

Though translation of OR findings into policy and practice is a complex and context sensitive process, interaction and trust between policymakers and researchers were found to be important factors in the use of research for policymaking, as were political and bureaucratic processes and the relevance or quality of the research. Efforts to support research findings translation in lower and middle income countries need to foster collaboration right from the beginning of the research. This can further improve local research capacity and full involvement of the community [5, 20, 22].

### Conclusion

This study revealed lack of positive behavioural change in conducting and acceptance of malaria interventions OR as a major bottleneck to addressing country specific MOR needs, followed by inadequate local funds. Adequate involvement of community members and health care workers is essential to improve as well as ascertain effectiveness and efficacy of interventions.

Identifying sources of domestic funds and its judicious use, strong advocacy for increased and sustained funding as well as effective and efficient use of existing resources to fill existing gaps needs to be maintained at all levels of malaria control.

## Limitations

The study relied on self-reported information which is prone to bias due to the background and personal interest of the respondents. This may be coupled with their awareness of the study objectives which cannot be excluded.

## Additional files

**Additional file 1.** Agenda-setting for operational research (national malaria elimination programme). A semi-structured questionnaire used to elicit information on personal biodata, area of expertise, experience in MOR in Nigeria, challenges, bottlenecks in MOR and needs to address the gaps identified.

**Additional file 2.** Working experience of the study participants on MOR and its associated challenges. A tabular data summarising the study participants’ working experiences and challenges faced while conducting MOR in Nigeria.

## Abbreviations

OR: operational research
MOR: malaria operational research
PSM: procurement and supply chain management
WHO: World Health Organisation
NMEP: National Malaria Elimination Programme
ACSM: advocacy, communication and social mobilisation
PM: programme management
CM: case management
MiP: malaria prevention
SME: surveillance, monitoring and evaluation
NMSP: National Malaria Strategic Plan
NFELTP: Nigeria Field Epidemiology And Laboratory Training Programme
