# Supplementary material for: Bottlenecks, concerns and needs in malaria operational research: the perspectives of key stakeholders in Nigeria
Source: BMC Res Notes. 2018 May 4;11:272. doi: 10.1186/s13104-018-3379-5 (PMC5935974; doi:10.1186/s13104-018-3379-5)
Supplement: Supplementary file 1 — Additional file 1. Agenda-setting for operational research (national malaria elimination programme). A semi-structured questionnaire used to elicit information on personal biodata, area of expertise, experience in MOR in Nigeria, challenges, bottlenecks in MOR and needs to address the gaps identified. [file 13104_2018_3379_MOESM1_ESM.doc]

**Additional file 1**

**Unique ID: ………..**

**AGENDA-SETTING FOR OPERATIONAL RESEARCH**

**(NATIONAL MALARIA ELIMINATION PROGRAMME)**

**Questionnaire**

The Nigeria Field Epidemiology and Laboratory Training Programme and National Malaria Elimination Programme welcome you to this survey that is being conducted as part of a study towards Setting Malaria Operational Research (OR) agenda in Nigeria. The aim is to get a wide consensus-based data for research agenda setting exercise. This is towards improving the efforts at moving malaria control in Nigeria forward.

As a Nigerian malaria researcher in-country, Nigerian researcher in diaspora, international researcher with interest in malaria control in Nigeria and funding partner in malaria research, you have been recognised to be an important stakeholder in malaria control. The information and advice you provide will assist us to gain a better understanding of the landscape of OR projects in malaria control and elimination, and to support OR agenda setting for malaria elimination in Nigeria.  We hereby humbly request your participation in this important survey.

Your decision to complete this survey is voluntary. We will maintain complete confidentiality and anonymity of your responses. It should take you about 15-20 minutes to complete this survey, which consists of 4 sections: general information, malaria operational research in Nigeria, operational research gaps and needs as well as comments.

I consent to participate

I do not consent Thank you, end survey

Name: …………. Signature Date…

Interviewer to detach from the main questionnaire and kept separately for confidentiality

**General Information Date: …………………..**

| **S/N** | **Question** | Provide your answers for each question in this column as appropriate |
| --- | --- | --- |
| **1** | **Age in years** |  |
| **2** | **Sex** | 1. Male 2. Female |
| **3** | **Area of Expertise** | Write the number corresponding to your response in box   1. Entomology 2. Parasitology 3. Clinical management 4. Therapeutics 5. Data management 6. Administrator 7. Policy and programme management 8. Others *(please specify):* …………………… |
| **4** | **How did you know about this survey?** | Write the number corresponding to your response in box   1. Email, 2. colleague, 3. in-person interview 4. Other *(please specify)*: ………………… |
| **5** | In which country do you **currently** work (if more than one list the one you're mostly based)? | __________________________ |
| **6** | Do you have **any** experience working on malaria in any other country? | 1. Yes 2. No |
| **7** | If 'yes', please list names of the country (ies) | 1. ____________  2._______________  3. ______________ |
| ***NOTE: [If No to Q6 and Q7, Skip to Q9]*** | | |
| **8** | What proportion of your malaria research work takes place in the following areas? | Provide the proportion (%) in the box   1. Academic (e.g. universities, research institutes), 2. Local implementation (e.g. government, local NGO, policymakers) 3. International implementation (e.g. international NGO) 4. Other *(please specify)*: ……………………………………………………. |

1. **Malaria operational research in Nigeria**

**Read definition:**

**Operational research is a form of research you conduct because you are looking for solutions to problems. Often when you look at a system or disease [e.g. malaria], you may find that things are not going the way you expect them to go (i.e. there are gaps and challenges), so you conduct a research to find out what the issues are and proffer solutions/interventions to that. The research can also ascertain the feasibility, effectiveness, accessibility and the coverage of the intervention(s)**

| **S/N** | **Questions** | Provide your answers for each question in this column as appropriate |
| --- | --- | --- |
| **9** | Based on your experience and familiarity with research literature, do you think that Nigeria’s malaria OR agenda should be different from malaria OR agenda in other settings? | 1. Yes  2. No  99. Don't know |
| **10** | If yes or no to Q9? Why? | ……………………………….. |
| **11** | Have you ever been involved in malaria Operational Research in Nigeria? | 1. Yes 2. No [if No, skip to Q25]   99. Don't know |
| **12** | If 'yes' to Q11, which aspect of malaria OR were you involved in? | **…………………………….**  **…………………………..** |
| **13** | What was the intervention (s) in (12)? | **…………………………..** |
| **14** | How was the intervention in (12) implemented? | **…………………………..** |
| **15** | Did you experience any challenge(s) in conducting the research? | 1. Yes 2. No   99. Don't know |
| **16** | If yes, What kind of challenge (s)? | 1.  2.  3. |
| **17.** | What influenced your choice of study topic? | 1.  2. |
| **18.** | Do you usually consult Nigeria National Malaria Elimination Programme’s (NMEP) list of priority research areas before deciding on which research to conduct? | 1. Yes 2. No |
| **19.** | If Yes, where did you get this priority list? | ……………………..  …………………….. |
| **20.** | If No, why? | ...................  ................... |
| **21.** | Has any of your malaria research work being shared? | 1. Yes 2. No |
| **22** | if ‘yes’, with whom? (Circle all that applies) | 1. University 2. National Malaria Elimination Programme (NMEP), Nigeria 3. Government agency/Ministries of Health 4. Funders 5. Media 6. Journals 7. Other(s) specify |
| **23.** | Did you receive any financial support for your malaria OR? | 1. Yes 2. No |
| **24.** | If ‘yes’, from who/agency [e.g WHO, Global fund, PMI] did you receive financial support? | ........................................................................  ........................................................................ |

1. **Malaria operational Research gaps and needs**

**Definitions**

**Research gaps/Challenges:** These are factors/bottlenecks (impediments) that limit the effective implementation of efforts of control of malaria, conduct of research to evaluate malaria interventions and innovative strategies to control of malaria. If solved, could enhance malaria control activities in the country. These could be related to health system, preventive and control measures, and community issues. E.g there is a lack of sensitive and specific tests for the diagnosis of malaria cases with low parasitemia; Low compliance to antimalarial drugs (side effects, taste, poor knowledge)

**Research Needs:** What is needed to be able to address the gaps effectively that is lacking. For example, need for increased budget, new technologies, surveillance tools, technical assistance and training for human capacity.

We are requesting you to **briefly outline what you think are the main research gaps/challenges** on malaria in Nigeria with regards to setting a robust and focused malaria operation research agenda and their **major causes/concerns.** This could be based on your experience or literature on malaria. The research gaps may cut-across the following thematic areas; *case management; prevention; surveillance, monitoring and evaluation; information, education and communication (IEC) and behaviour change communication (BCC); programme management*

- List at most **2 research gaps/challenges, major reasons and** research needs for each sub-theme in each thematic area in the tables below

- Please rank the mentioned **research gaps** in each sub-theme from 1 to 2 (1 for the research gap of higher priority)

Kindly state **the research needs that if made available will enable conduct of operations research in the different thematic areas**.

1. **Operational Research gaps/challenges, major reasons and research needs on malaria prevention**

| **Sub-theme** | **Research Gaps/Challenges/ bottlenecks** | **Major causes/concerns** | **Research needs** | **Rank priority gap (1-2)** |
| --- | --- | --- | --- | --- |
| Chemoprevention (e.g. Intermittent preventive therapy (IPTp), seasonal malaria chemoprevention (SMC etc.) |  |  |  |  |
|  |  |  |  |
| Insecticides |  |  |  |  |
|  |  |  |  |
| Long-lasting insecticidal nets (LLIN): availability, use, campaigns; Indoor residual spraying, etc. |  |  |  |  |
|  |  |  |  |
| Environmental management |  |  |  |  |
|  |  |  |  |
| Vector behaviour |  |  |  |  |
|  |  |  |  |

1. Research gaps/challenges, major reasons and research needs on malaria case management

| **Sub-theme** | **Research Gaps/Challenges/ bottlenecks** | **Major causes/concerns** | **Research needs** | **Rank priority gap (1-2)** |
| --- | --- | --- | --- | --- |
| Diagnosis |  |  |  |  |
|  |  |  |  |
| Treatment: availability of drugs, cost of drugs, drug quality, adherence to treatment guidelines etc. |  |  |  |  |
|  |  |  |  |

1. **Operational Research needs on surveillance, monitoring and evaluation**

| **Sub-theme** | **Research Gaps/Challenges/ bottlenecks** | **Major causes/concerns** | **Research needs** | **Rank priority gap (1-2)** |
| --- | --- | --- | --- | --- |
| Surveillance |  |  |  |  |
|  |  |  |  |
| Monitoring and evaluation |  |  |  |  |
|  |  |  |  |

1. Research gaps/challenges, major reasons and research needs on Information, education and communication (IEC) and behaviour change communication (BCC)

| **Sub-theme** | **Research Gaps/Challenges/ bottlenecks** | **Major causes/concerns** | **Research needs** | **Rank priority gap (1-2)** |
| --- | --- | --- | --- | --- |
|  |  |  |  |  |
|  |  |  |  |
|  |  |  |  |  |
|  |  |  |  |
|  |  |  |  |  |
|  |  |  |  |

1. Research gaps/challenges, major reasons and research needs on programme management

| **Sub-theme** | **Research Gaps/Challenges/ bottlenecks** | **Major causes/concerns** | **Research needs** | **Rank priority gap (1-2)** |
| --- | --- | --- | --- | --- |
| Funding |  |  |  |  |
|  |  |  |  |
| Policy |  |  |  |  |
|  |  |  |  |
| Timing and Planning implementation of malaria control activities |  |  |  |  |
|  |  |  |  |
| Coordination with partners |  |  |  |  |
|  |  |  |  |

1. Research gaps/challenges, major reasons and research needs on supply chain management

| **Sub-theme** | **Research Gaps/Challenges/ bottlenecks** | **Major causes/concerns** | **Research needs** | **Rank priority gap (1-2)** |
| --- | --- | --- | --- | --- |
| Supply chain management |  |  |  |  |
|  |  |  |  |

1. **Comments**
2. Before you complete the survey, do you have any other comments to share with us, with respect to the research gaps and needs you stated? *(Optional)*

*………………………………………*

*………………………………………*

*………………………………………*

1. **Next stage**

In the next stage of this study we will be developing and creating consensus to develop the final research agenda and if you are interested in being involved in this next stage to refine the agenda and plan implementation of the OR agenda questions, please write a mail to the contact address below to indicate this interest. [Do not write your contact detail here for confidentiality]

If you have any other questions, please contact us via: [agendasetting@nfeltp.org](mailto:agendasetting@nfeltp.org)

Thank you for completing this survey.
